# PI3K in stemness regulation: from development to cancer

**DOI:** 10.1042/BST20190778

**Published:** 2020-02-03

**Authors:** Ralitsa R. Madsen

**Affiliations:** UCL Cancer Institute, Paul O'Gorman Building, University College London, 72 Huntley Street, London WC1E 6DD, U.K.

**Keywords:** cancer, development, PI3K signalling, PIK3CA, pluripotent stem cells, stemness

## Abstract

The PI3K/AKT pathway is a key target in oncology where most efforts are focussed on phenotypes such as cell proliferation and survival. Comparatively, little attention has been paid to PI3K in stemness regulation, despite the emerging link between acquisition of stem cell-like features and therapeutic failure in cancer. The aim of this review is to summarise current known and unknowns of PI3K-dependent stemness regulation, by integrating knowledge from the fields of developmental, signalling and cancer biology. Particular attention is given to the role of the PI3K pathway in pluripotent stem cells (PSCs) and the emerging parallels to dedifferentiated cancer cells with stem cell-like features. Compelling evidence suggests that PI3K/AKT signalling forms part of a ‘core molecular stemness programme’ in both mouse and human PSCs. In cancer, the oncogenic *PIK3CA^H1047R^* variant causes constitutive activation of the PI3K pathway and has recently been linked to increased stemness in a dose-dependent manner, similar to observations in mouse PSCs with heterozygous *versus* homozygous *Pten* loss. There is also evidence that the stemness phenotype may become ‘locked’ and thus independent of the original PI3K activation, posing limitations for the success of PI3K monotherapy in cancer. Ongoing therapeutic developments for PI3K-associated cancers may therefore benefit from a better understanding of the pathway's two-layered and highly context-dependent regulation of cell growth *versus* stemness.

## An unsolved puzzle

**Figure 1.**
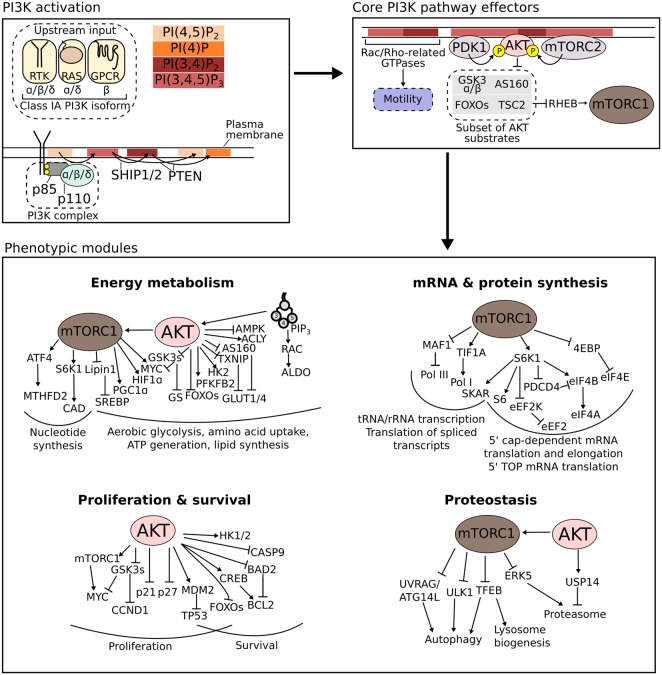
An overview of class IA PI3K signalling. The class IA PI3K heterodimer consists of one of three different catalytic subunits (p110α/β/γ) and one of five different regulatory subunits (p85α/β, p55α/γ, p50α). Its activation involves recruitment to the plasma membrane where its substrate, the phosphoinositide PI(4,5)P_2_ is located. A common mechanism of activation involves the binding of the regulatory p85 subunit to phosphotyrosine residues on receptor tyrosine kinases (RTKs) or their associated adaptor proteins. The catalytic subunits of PI3Kα and PI3Kδ can also interact and be activated by RAS. This is not the case for PI3Kβ which instead can be activated by other small GTPases downstream of G protein-coupled receptors (GPCRs). The immediate output of class IA PI3K activation is the generation of the second messenger PI(3,4,5)P_3_ and its derivative PI(3,4)P_2_. These are detected by proteins with specialised phosphoinositide-binding domains, with AKT representing one of the most studied examples. Through AKT-dependent and -independent effectors, the PI3K pathway orchestrates an array of diverse phenotypic modules whose execution is highly context-dependent [8]. Negative feedback loops and cross-talk with other pathways are omitted for clarity. For comprehensive PI3K signalling reviews, the reader is referred to Ref. [1,2,4,113].

Development and cancer can be described as two sides of the same coin, with cancer cells progressively co-opting and corrupting embryonic processes to support tumour growth and metastasis. The class IA phosphoinositide 3-kinase (PI3K) pathway is among the best studied in human biology, and its pathological hyperactivation is considered a ‘driver’ in numerous cancers as well as benign, developmental overgrowth [1]. The last two decades have provided a detailed mechanistic understanding of how this pathway regulates fundamental cellular processes such as survival, proliferation, migration and metabolism [2] (Figure 1). Accumulating evidence also suggests an important role for PI3K signalling in the regulation of stemness, yet the underlying mechanisms remain largely enigmatic.

Given the emerging link between cancer stemness and disease progression, a better mechanistic understanding of how the PI3K pathway impinges on critical developmental processes — either in forward (normal development) or reverse (cancer) mode — will be important for continued therapeutic development for PI3K-associated cancers. Collaterally, such research may also improve our understanding of key embryonic processes operating at early stages of developmental PI3K-related overgrowth disorders. Finally, insight into PI3K-dependent stemness regulation is likely to inform current efforts to establish improved stem cell culture protocols in developmental biology and regenerative medicine.

The aim of this review is to provide an overview of PI3K signalling in stemness regulation, with a focus on pluripotent stem cells (PSCs) and emerging parallels to cancer cells with stem cell-like properties. The need for a better mechanistic understanding of context-dependent PI3K-mediated stemness is highlighted, alongside the potential for systems biology and interdisciplinary approaches to gain insight into these important questions.

## PI3K signalling: the pathway that seems to do it all

Class IA PI3Ks are heterodimers of a regulatory (p85) and a catalytic (p110) subunit, with the resulting complexes referred to as PI3Kα, PI3Kβ and PI3Kδ based on the identity of the catalytic subunit (Figure 1). Among these, the ubiquitously expressed PI3Kα is essential for organismal growth and survival, with pleiotropic functions ranging from control of tissue patterning, angiogenesis and insulin-dependent metabolic regulation. Activating mutations in *PIK3CA*, the gene encoding the catalytic p110α subunit of PI3Kα, are also considered disease-drivers in human cancers as well as developmental overgrowth disorders known as PROS (*PIK3CA*-related overgrowth spectrum) [3].

Irrespective of the exact enzymatic complex, the primary output of PI3K activation is the production of the second messenger phosphatidylinositol-3,4,5-trisphosphate (PIP_3_) and its dephosphorylated derivative PI(3,4)P_2_. Among their key effectors are the three serine/threonine kinase AKT isoforms, which control the activity of major cellular proteins, including the glycogen synthase kinase 3 isoforms (GSK3α/β), forkhead box O (FOXO) transcription factors and mechanistic target of rapamycin complex 1 (mTORC1) [4]. Through modulation of the actin cytoskeleton, PI3K activity also regulates multiple AKT-independent nodes, including those involved in membrane ruffling and cell migration [5] (Figure 1). Given this ability to impinge on critical cellular processes, the PI3K pathway is subject to exquisite control, including multiple negative feedback loops [1,4] and direct inactivation by several lipid phosphatases, most notably by the ubiquitously expressed tumour suppressor phosphatase and tensin homologue (PTEN) which 3-dephosphorylates both PIP_3_ and PI(3,4)P_2_ [6,7]. PI(3,4)P_2_ is generated by the 5-phosphatases SHIP1 and/or SHIP2, which are found both on the plasma membrane and the early endosomal compartments, thus contributing to the spatial regulation of phosphoinositide signalling [2].

While PI3K signalling might be seen as capable of regulating most major cellular processes (Figure 1), its output is usually rather specific and highly context-dependent — governed by cell-specific gene expression programmes, signalling thresholds and environmental context [8]. Considering PI3K signalling as a ‘pathway’ is itself a simplification used to conceptualise a complicated network of signalling components. In reality, PI3K signalling components cross-talk with effectors of other major pathways, including those of RAS/MAPK [9,10], WNT/β-catenin [11,12], NF-κB [13] and TGFβ [14–17]. The resulting complexity presents a significant challenge for conventional reductionist approaches and, consequently, remains poorly understood, with most studies focussing on isolated PI3K signalling effects.

## Cancer: ‘reverse’ development

There are numerous mechanisms through which normal cells may acquire malignant features [18]. A common feature, however, is the convergence on a phenotypic programme with aberrant access to cellular functions with key roles in embryogenesis and tissue self-renewal [19]. Characteristics such as replicative immorality, lineage plasticity and the ability to undergo epithelial-to-mesenchymal transition (EMT) are shared between cancer cells and the PSCs that orchestrate early embryonic development [20,21]. Accordingly, embryonic markers such as NANOG, OCT3/4 and SOX2 are re-expressed across different human cancers and have been linked to poor clinical outcome [22]. Furthermore, a recent systems-level analysis of 17 major cancer types identified up-regulation of cell growth genes and the down-regulation of differentiation genes as a general pattern associated with shorter patient survival [23].

There is also ample evidence for a link between the acquisition of stemness properties and therapeutic resistance in cancer [24,25]. Stemness features in tumours are attributed to the presence of a subpopulation of cancer stem cells (CSCs), defined as cells with high self-renewal capacity and the ability to regenerate the heterogeneity of the primary tumour in functional experimental assays [25]. Understanding the molecular mechanisms that stabilise the CSC state, and that set it apart from the bulk of the remaining tumour cells, is therefore critical for effective therapeutic targeting [24].

## PI3K signalling in cancer stemness

The PI3K pathway is frequently hyperactivated across multiple human cancers, either due to direct genetic and/or epigenetic dysregulation of pathway effectors or indirectly, due to aberrant signalling inputs (e.g. hyperactivation of upstream receptors, loss of negative feedback regulation). In particular, activating mutations in *PIK3CA*, the gene encoding the catalytic subunit of PI3Kα, are among the most common across multiple human cancer types and are also the cause of benign yet highly debilitating developmental overgrowth disorders [3].

Expression of the most frequently occurring *PIK3CA* cancer hotspot variant, H1047R, has been linked to dedifferentiation and stemness in mouse models of breast [26–28], lung [29] and colorectal [30] cancers. A similar phenotype has been observed upon wild-type *PIK3CA* overexpression in a murine head and neck cancer model [31]. Nevertheless, the exact molecular mechanism(s), including an often-reported requirement for additional oncogenic hits, have remained elusive. It is noteworthy that heterozygous *PIK3CA^H1047R^* on its own rarely suffices to induce cancer in mice [32], consistent with the benign disease in individuals with congenital *PIK3CA*-related overgrowth [3]. Interestingly, vascular malformations represent one of the most common and debilitating phenotypes in the *PIK3CA*-related overgrowth spectrum (PROS), and when modelled in mice, these lesions exhibit loss of arteriovenous identity markers, suggesting lineage identity loss and dedifferentiation even in some benign disease settings [33].

Oncogenic PI3Kα activation has also been linked with induction of EMT [28,30,34–36] — a process that is itself characterised by enormous plasticity and multiple intermediate states [24,37]. The connection between cancer stemness and EMT is suggested to hinge upon induction of autocrine signalling loops, including those involving the pro-tumorigenic action of the TGFβ pathway [24,38]. Given compelling evidence for a link between TGFβ and PI3K signalling in regulation of stemness in cancer-relevant cell models [31,35,39], as well as the involvement of both pathways in developmental stemness (see below), it will be important for future studies to determine whether cancer cells co-opt the developmental functions of the two pathways to acquire stemness properties that are associated with therapeutic resistance.

Such studies are inherently difficult to perform because CSCs are thought to represent rare cell populations in most tumours [25]. They may be enriched by using *in vitro* cancer spheroid models [40], but will still fall short of capturing the evolution of the stemness phenotype upon induction of oncogenic PI3K signalling in otherwise normal cells. While adult stem cells may represent an alternative option, hyperactivation of PI3K signalling in these cells is often associated with stem cell exhaustion and terminal differentiation (see section ‘A note on context'). For instance, homozygous *Pten* loss in mice leads to depletion of haematopoietic stem cells (HSCs), but promotes the generation of their transformed counterparts — leukemic stem cells [41]. This suggests that studies of PI3K-dependent stemness regulation in the context of cancer progression will benefit from availability of non-transformed cell lines that are nevertheless capable of unlimited self-renewal in the face of oncogenic pathway activation. Normal PSCs are characterised by a diploid genome and lack of oncogenic mutations, yet are naturally immortal and exhibit many phenotypic parallels to cancer cells [20,21]. This provides an opportunity to use PSCs as a model system to study PI3K pathway-dependent regulation of stemness, with subsequent testing of relevant findings in bona fide CSCs.

## PI3K signalling in developmental stemness

### PSC primer: the importance of species and developmental stage

PSCs such as mouse and human embryonic stem cells (mESCs and hESCs, respectively), or the corresponding induced pluripotent stem cells (iPSCs), are capable of multilineage differentiation and can theoretically give rise to any cell type in the adult organism (Box 1).

**BOX 1: The many faces of stemness**

‘Stemness’ is used to describe lack of differentiation or partial dedifferentiation and is typically applied in studies of stem cells, yet the definition of a stem cell is itself dependent on the particular context under study. Mammalian development starts with a fully undifferentiated single cell known as the totipotent zygote (see Figure below). This ultimate state of stemness is transient and quickly gives rise to the two cell lineages that define the developing blastocyst — the inner cell mass and the trophectoderm. The inner cell mass consolidates into the pluripotent epiblast from which all future embryonic lineages develop [130]. While short-lived *in vivo*, PSCs can be isolated and propagated indefinitely under the right conditions *in vitro*, thus forming the basis for the so-called human embryonic stem cells (hESCs). In this context, ‘stemness’ refers to the indefinite self-renewal of the undifferentiated cells, while pluripotency denotes their ability to differentiate to derivatives of the three embryonic germ layers (ectoderm, mesoderm, endoderm) [131].

At the other end of the spectrum, fully differentiated cells can acquire ‘stemness’ properties through the process of partial or complete dedifferentiation as seen in cancer or during the process of artificial reprogramming of somatic cells into induced pluripotent stem cells (iPSCs). Somewhere in between these two lie multipotent, bipotent and unipotent adult stem cells which are relatively differentiated yet capable of self-renewal and additional specification into tissue-specific cell types (Figure below). Neural stem cells (NSCs), mesenchymal stem cells (MSCs) and HSCs are examples of multipotent stem cell types, whereas more tissue-specific stem cells such as mammary stem cells or intestinal stem cells are more limited in their differentiation capacity.

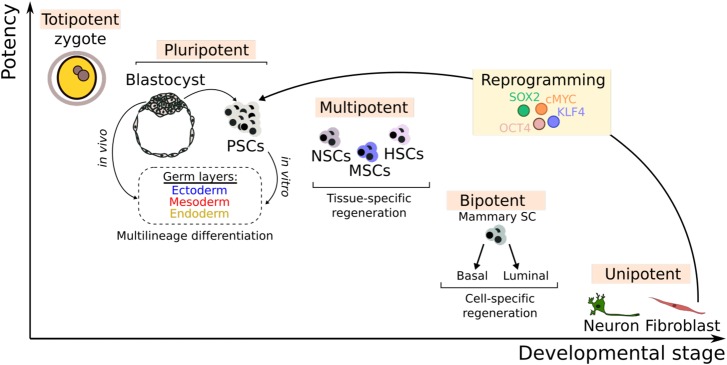

Comparisons of mouse and human PSCs have revealed critical differences, including the timing of transcriptional activation of the embryonic genome, distinct mechanisms to achieve X-chromosome dosage compensation in female lines, as well as differences in the configuration of signalling networks (reviewed in Ref. [42–45]). In addition to species divergence, these differences reflect the *in vitro* stabilisation of two distinct developmental states — the naïve pre-implantation state for mESCs and the primed post-implantation state for human PSCs (hPSCs) [43,46].

Differences between mouse and hPSCs are particularly important to consider when it comes to extrapolation of mechanistic insights from one system to the other. The core pluripotency gene regulatory network is a pertinent example. While co-ordinated by the same highly conserved transcription factors — NANOG, SOX2, OCT3/4 — in both mouse and hPSCs, downstream target gene regulation is poorly conserved [43,47,48]. More generally, this illustrates a recurrent point in this review — the notion that the same set of signalling effectors can be plugged into different regulatory layers in different cell types.

### PI3K-induced stemness in mouse and human PSCs

Although several of the major cell signalling pathways, including MAPK/ERK and WNT, have opposing effects on mouse *versus* hPSCs [49,50], both cell systems exhibit a consistent reliance on PI3K signalling not only for survival but also for sustained stemness. This suggests that the PI3K pathway forms part of a ‘core molecular stemness programme’ in PSCs [51], with the underlying signalling network undergoing substantial remodelling in response to differentiation signals [52].

The strongest evidence for PI3K-induced stemness comes from genetic perturbations that result in constitutive activation of the pathway. This was initially achieved through *Pten* ablation in mESCs, resulting in their impaired differentiation both *in vitro* and *in vivo*[53]. The effect of *Pten* loss is allele dose-dependent, with *Pten^−/−^* mESCs giving rise to large, undifferentiated tumours *in vivo*, whereas their heterozygous counterparts generate well-differentiated tumours composed of tissues from all three embryonic germ layers [53]. Such tumours are known as teratomas and the capacity to form them is used to test for pluripotency. Therefore, homozygous but not heterozygous *Pten^−/−^* mESCs exhibit sustained stemness alongside impaired pluripotency. Although a subsequent study using a different mouse strain did not observe alternations in differentiation capacity between *Pten^−/−^* mESCs and wild-type counterparts, once differentiated, a subset of *Pten*-null mESC derivatives failed to down-regulate *Nanog* and *Oct3/4* expression, resulting in a greater capacity for tumour formation [54]. These results are consistent with residual stemness and impaired pluripotency in a subset of *Pten^−/−^* mESCs. Similar to mESCs, hESCs with *PTEN* knock-down exhibit increased self-renewal and up-regulated expression of *NANOG* and *OCT3/4*, in conjunction with activation of canonical PI3K/AKT signalling and resistance to multilineage differentiation in three-dimensional (3D) embryoid body (EB) assays *in vitro* [55].

Given that PTEN can have PI3K-independent effects [56], other studies have investigated the link between PI3K pathway activation and stemness more directly by modulating key pathway effectors. In mESCs and primate PSCs, overexpression or constitutive activation of AKT results in self-sustained stemness, characterised by persistent expression of PSC markers and impaired differentiation *in vitro* [57,58]. More recently, an allelic series of isogenic hPSCs with endogenous heterozygous or homozygous expression of the PI3Kα-activating cancer-driver mutation *PIK3CA^H1047R^* were shown to exhibit a striking allele dose-dependent stemness phenotype [59] — similar to the aforementioned findings with heterozygous *versus* homozygous loss of *Pten* in mESCs. Thus, homozygous but not heterozygous *PIK3CA^H1047R^* mutants were characterised by self-sustained stemness both *in vitro* and *in vivo*, accompanied by graded activation of the PI3K pathway, partial loss of epithelial morphology and widespread transcriptional remodelling with up-regulated expression of multiple PSC markers, including *NANOG* and *OCT3/4* [59]. The stemness phenotype of *PIK3CA^H1047R/H1047R^* hPSCs is similar to previous observations in mESCs with enhanced AKT activation [57,58] or GSK3α/β double knock-out [60], as well as to mouse and monkey PSCs expressing membrane-targeted and thus constitutively active PDK1 (3-phosphoinositide-dependent protein kinase 1; PDPK1) or AKT [57,61]. Combined, these studies suggest that above a certain threshold, constitutive PI3K activation leads to AKT-dependent self-sustained renewal of PSCs.

A different question is whether physiological levels of PI3K activation are required for continuous maintenance of PSCs. Work in this area has primarily been carried out in mESCs, and caution is warranted before extrapolating the proposed mechanisms to hPSCs (see section ‘*Pluripotent stem cell (PSC) primer: the importance of species and developmental stage')*. Such limitations notwithstanding, genetic and pharmacological studies by the Welham group have demonstrated that PI3K signalling is required for maintenance of the undifferentiated state in mESCs [62–64]. Conversely, knock-down of *Akt1* leads to loss of mESC self-renewal [65,66]. Pharmacological PI3K inhibition in hPSCs has also been linked to increased differentiation [67–71], yet the evidence is mainly based on the use of the pan-PI3K inhibitors, LY294002 and wortmannin, which are known to be promiscuous towards multiple other kinases — including mTOR — at the applied concentrations [72–76]. The use of these inhibitors is strongly discouraged by experts in the PI3K signalling field [4].

## Mechanistic insights: the known unknowns

Despite substantial evidence that PI3K signalling promotes stemness in PSCs, the underlying mechanisms have yet to be defined [77]. From receptor activation to the specific PI3K isoform(s) and its downstream effectors, the exact sequence of events and their contribution to PSC phenotypes warrant more systematic studies. The following is an attempt to summarise the known unknowns and thus facilitate the generation of novel hypotheses for future studies in this area.

### Receptor-mediated PI3K activation

**Figure 2.**
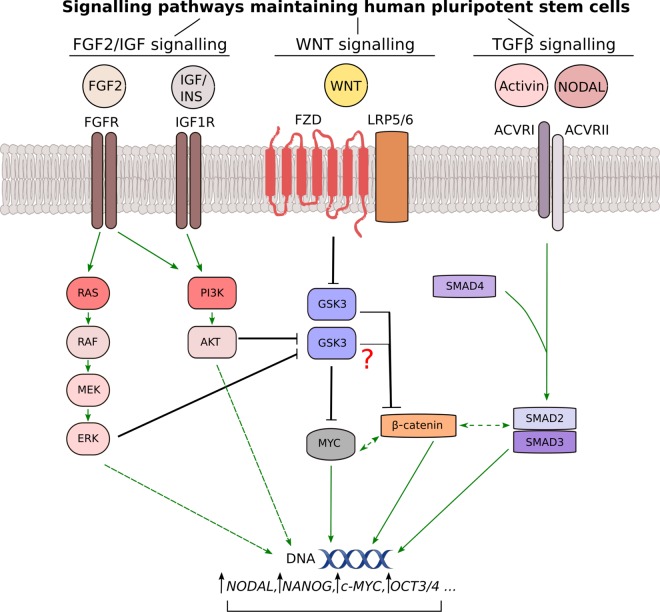
Core signalling pathways maintaining human pluripotent stem cells (hPSCs). The pluripotent state is inherently unstable and minor perturbations disrupting the balance within the signalling network may lead to the initiation of differentiation to either one of the three germ layers or to extraembryonic derivatives. As a consequence, the shown pathways may act both to promote stemness in one setting and differentiation in another, all depending on the microenvironmental context, as well as the subcellular localisation and signalling dynamics of individual pathway components. Note that several of the displayed effectors exist in multiple isoforms and are currently omitted for clarity, although there are cases in which two isoforms may have different or even opposing effects on PSC biology [143]. The red question mark is used to denote existing uncertainty about the ability of PI3K and WNT signalling to access the same GSK3 pool. Dashed lines are used to indicate indirect regulatory relationships. Positive regulation is shown in green and negative regulation in black.

Advances in regenerative medicine have long called for more defined culture conditions for hPSCs, including coating substrate and growth medium. At present, the most widely used media solutions in the field are the commercially available mTESR1 and Essential 8/E8, with the latter allowing cells to be cultured in DMEM/F12 supplemented with only eight components [78]. Three of these eight components — insulin, FGF2 and TGFβ (or its alternative, NODAL) — represent growth factors/cytokines that are critical for hPSC survival and continued self-renewal (Figure 2) [78–86]. Insulin is well known to act in a PI3K-dependent manner, but is also able to induce activation of the mitogenic MAPK/ERK pathway [87]. FGF2 is a potent inducer of MAPK/ERK and can also activate PI3K. Further complexity emerges from the context-dependent cross-talk between the two pathways [88], either directly or indirectly. It is, however, unclear to what extent each growth factor leads to activation of one pathway over the other, and whether a specific balance needs to be attained for continuous PSC self-renewal — as observed in endothelial regulation of HSC maintenance [89]. Beyond FGF2 and insulin, whether TGFβ pathway activation promotes PI3K signalling in hPSCs — as reported in other contexts [14,17,90–92] — requires further and more systematic investigation. The available evidence remains inconclusive and may reflect differences in culture conditions and examined signalling time points [69,93].

Additional inputs into the PI3K pathway are also known to arise from autocrine and paracrine signals such as the endogenously secreted peptide ELABELA [94]. Finally, mechanotransduction is closely intertwined with both PI3K and MAPK/ERK signalling [95], and it is conceivable that differences in coating substrate may alter the dependencies on one or several of the aforementioned growth factors when it comes to PI3K activation. PI3K pathway activation is itself linked to altered expression of extracellular matrix components, at least in mESCs and their derivatives [96,97].

### The specific PI3K isoform

At the level of the PI3K heterodimer itself, very few studies have attempted to determine the identity of the main PI3K catalytic isoform(s) responsible for pathway activation in PSCs. Treatment of hPSCs with a relatively low-dose (100 nM) of the PI3Kα-specific inhibitor BYL719 reduces AKT phosphorylation both at baseline and in response to different growth factors, whereas treatment with TGX221 at a dose (500 nM) that would inhibit both PI3Kβ and PI3Kδ has no effect [58,98,99]. These findings agree with the identification of *PIK3CA* as an essential gene in a recent knock-out CRISPR screen in haploid hPSCs [100]. In contrast with findings in mESCs [64], however, PI3Kα and not PI3Kβ so far appears to be the main isoform responsible not only for promoting survival but also stemness in hPSCs [58,98,99].

### Downstream effectors

The exact signalling mechanisms whereby activation of PI3K signalling leads to increased expression of stemness markers are the least well understood, particularly when it comes to hPSCs (for an mESC-focussed review on PI3K signalling, see Ref. [101]). Well-studied AKT-dependent PI3K pathway effectors with known roles in stemness include GSK3 and MYC. MYC represents a central hub in stemness regulation via its pleiotropic roles on the transcriptome and epigenome of PSCs [102], and is also one of the four Yamanaka factors used to reprogramme somatic cells to iPSCs [103]. GSK3-mediated phosphorylation of MYC primes this transcription factor for degradation [104], thus AKT-dependent inhibition of GSK3 downstream of PI3K activation would be expected to have the opposite effect. While this mechanism appears to operate in mESCs [105], its importance in hPSCs has been disputed [93,106]. PI3K signalling can also lead to increased MYC levels through a translational mechanism that relies on mTORC1 activation [107], but this has yet to be studied in a stem cell context.

PI3K-dependent GSK3 inhibition may also lead to direct stabilisation of β-catenin, although the extent of this cross-talk remains subject to debate and may reflect indirect transcriptional changes through MYC or other effectors, as opposed to rapid post-translational regulation [108]. Among its many transcriptional targets, β-catenin — a downstream effector of canonical WNT signalling — promotes *NODAL* expression and thus TGFβ signalling, with both WNT and TGFβ pathways known to function in a dose- and time-dependent manner in developmental biology. It remains to be determined whether the recent discovery of dose-dependent stemness regulation downstream of oncogenic PI3K activation in hPSCs features an initial β-catenin-driven enhancement of *NODAL* expression and subsequent induction of self-sustained stemness, in a manner that is strictly dependent on a particular threshold of PI3K pathway activation (Box 2).

**BOX 2: *PIK3CA^H1047R^* dose-dependent effects on stemness**

Mainly performed in mouse ESCs treated with LY294002 or expressing dominant-negative PI3K regulators, some early studies linked PI3K activity to increased *Nanog* expression through a PI3Kβ-dependent but AKT-independent mechanism [64,132,133]. More recently, specific perturbation of PI3Kα by both genetic and pharmacological means revealed a previously unknown link between activation of this enzyme and acute dose- as well as time-dependent regulation of *NODAL* expression, prior to any changes in *NANOG* [58,99] — a well-known transcriptional target of TGFβ/NODAL signalling [134]. Furthermore, homozygous *PIK3CA^H1047R^* hPSCs no longer require continuous PI3K pathway activation to sustain the enhanced stemness gene signature, consistent with their autocrine activation of TGFβ/NODAL signalling [99]. Evidence from bona fide cancer models of oncogenic PI3K pathway activation also suggests that the stemness phenotype can become uncoupled from the original trigger and thus no longer reversible simply through PI3K inhibition [99,135,136].

Exactly how the PI3K pathway controls *NODAL* expression in a dose-dependent manner remains unknown. One candidate worthy of further investigation is β-catenin due to its ability to activate transcription of *NODAL*, with NODAL subsequently sustaining its own expression through an autoregulatory positive feedback loop [137]. There is some evidence for an interaction between WNT/β-catenin and oncogenic PI3K pathway activation in promoting intestinal [30], mammary [138] and leukemic [135] stem cell maintenance, yet further studies will be required to determine the nature of this cross-talk and whether it operates in response to *PIK3CA^H1047R^* expression in hPSCs.

Investigation of a potential link to the MYC oncogene is also warranted given its prominent role as a hub gene in computational network analyses of homozygous *PIK3CA^H1047R^* hPSCs [99]. Conditional MYC activation in mESCs has been shown to establish a self-sustained stemness phenotype which ultimately becomes independent of the presence of MYC activation [139], similar to the inability of PI3Kα inhibition to reverse the self-sustained stemness phenotype in homozygous *PIK3CA^H1047R^* hPSCs [99]. It is also noteworthy that MYC's biological effects have been linked to distinct thresholds of abundance [140]. Finally, *PIK3CA^H1047R^* was recently shown to co-operate with oncogenic *KRAS* in promoting MYC activity and tumorigenesis in mammary breast epithelial cells [141], a cellular system in which a link between oncogenic PI3K signalling and stemness has been demonstrated [35,142].

Others have suggested an alternative model linking PI3K, GSK3 and TGFβ signalling to explain the stemness-promoting ability of the PI3K pathway in hPSCs [93]. According to this model, PI3K activation indirectly promotes GSK3 activity and thus inhibits WNT/β-catenin signalling, which serves to keep TGFβ signalling below the activity threshold required for mesendodermal differentiation [93]. This indirect mechanism relies on PI3K-dependent inhibition of ERK, thus relieving ERK's inhibitory phosphorylation of GSK3 on the same Serine residue that is also known to be phosphorylated by AKT [93]. However, the supporting evidence is based on the use of the relatively non-specific inhibitors, LY294002 and PI-103, thus warranting additional confirmation. Arguing against this mechanism, GSK3 phosphorylation is ablated in response to PI3Kα-specific inhibition in wild-type as well as *PIK3CA^H1047R^* hPSCs, irrespective of the elevated ERK phosphorylation seen in *PIK3CA* mutant cells [99].

Another potential mechanism whereby PI3K signalling may promote stemness involves alteration of cellular metabolism and ‘knock-on’ effects on epigenetic regulation. The cancer field has contributed tremendous insight into how PI3K pathway activation alters major metabolic fluxes, most notably those associated with glycolysis, the citric acid cycle and lipid synthesis. This is closely linked with altered levels of key metabolites acting as substrates for chromatin- and DNA-modifying enzymes [109]. One well-studied example is the AKT-dependent increase in Acetyl-CoA levels in cells with hyperactive PI3K signalling, which in turn results in enhanced histone acetylation [110]. Conversely, a recent study demonstrated that Acetyl-CoA and the associated increase in histone acetylation sustains the stemness phenotype of hPSCs [111]. Moreover, MYC has been suggested to orchestrate the metabolic phenotype of hPSCs [112], although this has not been studied specifically in the context of PI3K activation and epigenetic changes. Thus, future studies are warranted to determine to what extent PI3K-induced stemness reflects metabolic regulation of the epigenome.

### A note on context

An important point about the aforementioned AKT-regulated PI3K pathway effectors is that they are all subject to regulation by other components beyond those involved in PI3K signalling. The relative contribution of individual inputs is likely dependent on the exact cellular state. In the case of mTOR, additional complexity arises from its incorporation into two different complexes (mTORC1 and mTORC2) and their involvement in an array of cellular signalling pathways [113]. Moreover, mTORC1 and its effector ribosomal S6 kinase comprise a negative feedback loop that limits upstream PI3K activity [113], thus resulting in a non-linear relationship between PI3K and mTORC1 activation. Currently, knowledge about the effect of mTOR activity on stemness in hPSCs remains unclear [114,115], though recent data suggest the existence of dose-dependent regulation [116]. The context-dependent wiring of the signalling network and its dynamic properties should also be considered when assessing the mechanisms behind the dose-dependent effects on stemness following *PTEN* inactivation or oncogenic *PIK3CA* expression in PSCs. The similarity of the cellular phenotypes, notwithstanding, PI3K pathway activation in *PTEN*-null cells remains dependent on external ligand stimulation, whereas strongly activating *PIK3CA* mutations (e.g. H1047R) also promote constitutive ligand-independent PI3K signalling [3]. Such differences could give rise to altered signalling dynamics and feedback regulation [8], which in turn may impinge on stemness regulation through distinct mechanisms.

While PI3K pathway activation seems to promote stemness in PSCs, this ability is not universal when it comes to adult stem cells, reflecting not only different stem cell niches and developmental timings, but also differences in differentiation stage among stem cells residing within the same niche. Given multiple reports of adult stem cell exhaustion or differentiation in response to oncogenic activation of the PI3K pathway [41,117–121], and in particular mTORC1 activation (reviewed in Ref. [122]), it will be important for future studies to determine the exact factors that allow the same set of pathway components to promote stemness in one setting but not in another. Similarly, continued studies of PSCs and their derivatives may also help explain the apparent lineage skewing and relative lack of increased cancer risk in overgrowth patients with the embryonic acquisition of otherwise highly oncogenic *PIK3CA* mutations [3]. This also calls for a better understanding of the potential contribution from non-cell-autonomous mechanisms, driven by interactions between *PIK3CA* mutant and wild-type cells in a mosaic context [3]. Precedents for such mechanisms already exist, with previous work demonstrating that the enhanced stemness of HSCs in mice with germline *Ship1* loss is a non-cell-autonomous consequence of an altered haematopoietic niche [123].

## Future directions

The medieval proverb ‘All roads lead to Rome' can conveniently be superimposed on to the current picture of PI3K-dependent stemness regulation. While the puzzle remains unsolved, efforts are made to approach it from multiple, perhaps even diametrically opposite, ways. This, in turn, can result in confusion and give the impression of inconsistent findings. Although true inconsistencies do occur – often owing to the use of non-specific approaches (e.g. the widespread application of non-specific PI3K inhibitors in the PSC field) — the vast majority are likely to reflect the true complexity of the phenomenon under study and the limited ability of conventional approaches to capture the cellular system in its entirety.

Recent technological advances in high content imaging and -omics technologies are offering novel ways in which future studies may address the complexity of PI3K-mediated stemness, through a combination of conventional mechanistic studies and emerging systems biology strategies applied successfully in other areas [124–127]. To succeed in providing a unifying picture of PI3K-mediated stemness in development and cancer, such systems biology approaches will necessitate better interdisciplinary ‘cross-talk’ to combine the multifaceted mechanistic data on this pathway already available into comprehensive computational models. The power of these models lies in their ability to handle the complexity of temporal parameters, signalling thresholds and combinatorial pathway interactions. This, in turn, allows for the generation of mechanistic predictions for otherwise poorly understood signalling phenomena. These predictions can subsequently undergo formal testing by conventional approaches and the results used to refine and improve the original models in what may be considered a cycle of continuous reiteration.

A mathematical model of PI3K signalling in hPSCs has been developed and used to study the pathway's information transmission principles in this particular context [128,129]. This model may serve as a starting point for further refinement based on prior and future data, potentially enabling previously intractable questions to be addressed: How are different doses and patterns of PI3K activation sensed and decoded by hPSCs as a function of genetic background and environmental context? Are similar decoding principles shared by CSCs, thereby allowing hPSCs to be used as a valid *in vitro* model system for an otherwise rare subpopulation of therapeutically relevant cancer cells? Are there selective vulnerabilities whereby inhibition of PI3K-induced stemness can be achieved without knock-on effects on essential functions such as metabolic regulation?

Answering these fundamental questions is valuable in its own right and may also inform further therapeutic development for PI3K-associated disorders. Undoubtedly, solving the ‘PI3K-stemness puzzle’ will be an investment with many returns.

## Perspectives

- Given the link between cancer stemness and therapeutic relapse, understanding the PI3K pathway's two-layered regulation of growth *versus* stemness is an important task for the future. Beyond its direct translational value, this understanding may further efforts to develop improved PSC culturing protocols in developmental biology and regenerative medicine.
- Oncogenic PI3K pathway activation has been linked to enhanced stemness in both cancer models and PSCs. At least in some contexts, this link appears to be exquisitely dependent on the dose of oncogenic PI3K signalling, yet the underlying mechanisms remain obscure.
- Solving the ‘PI3K-stemness puzzle’ will hinge upon adoption of emerging systems biology approaches, including computational models capable of handling the context-dependent regulation of the phenomenon under study. For such approaches to succeed, there is a need for greater cross-talk between the fields of cancer, signalling and developmental biology.

## Abbreviations

CSCs: cancer stem cells
EB: embryoid body
EMT: epithelial-to-mesenchymal transition
FOXO: forkhead box O
HSCs: haematopoietic stem cells
iPSCs: induced pluripotent stem cells
MSCs: mesenchymal stem cells
NSCs: neural stem cells
PIP_3_: phosphatidylinositol 3,4,5-trisphosphate
PROS: *PIK3CA*-related overgrowth spectrum
PSCs: pluripotent stem cells
PTEN: phosphatase and tensin homologue

## Open Access

Open access for this article was enabled by the participation of University College London in an all-inclusive *Read & Publish* pilot with Portland Press and the Biochemical Society under a transformative agreement with JISC.
